# Spectrally Matched Near-Threshold Noise for Subjective Tinnitus Loudness Attenuation Based on Stochastic Resonance

**DOI:** 10.3389/fnins.2022.831581

**Published:** 2022-03-30

**Authors:** Konstantin Tziridis, Sarah Brunner, Achim Schilling, Patrick Krauss, Holger Schulze

**Affiliations:** Experimental Otolaryngology, University of Erlangen-Nuremberg, Erlangen, Germany

**Keywords:** tinnitus treatment, low intensity acoustic noise, stochastic resonance, individualized medicine, tinnitus questionnaires

## Abstract

Recently, we proposed a model of tinnitus development based on a physiological mechanism of permanent optimization of information transfer from the auditory periphery to the central nervous system by means of neuronal stochastic resonance utilizing neuronal noise to be added to the cochlear input, thereby improving hearing thresholds. In this view, tinnitus is a byproduct of this added neuronal activity. Interestingly, in healthy subjects auditory thresholds can also be improved by adding external, near-threshold acoustic noise. Based on these two findings and a pilot study we hypostatized that tinnitus loudness (TL) might be reduced, if the internally generated neuronal noise is substituted by externally provided individually adapted acoustic noise. In the present study, we extended the data base of the first pilot and further optimized our approach using a more fine-grained adaptation of the presented noise to the patients’ audiometric data. We presented different spectrally filtered near-threshold noises (−2 dB to +6 dB HL, 2 dB steps) for 40 s each to 24 patients with tonal tinnitus and a hearing deficit not exceeding 40 dB. After each presentation, the effect of the noise on the perceived TL was obtained by patient’s response to a 5-scale question. In 21 out of 24 patients (13 women) TL was successfully subjectively attenuated during acoustic near-threshold stimulation using noise spectrally centered half an octave below the individual’s tinnitus pitch (TP). Six patients reported complete subjective silencing of their tinnitus percept during stimulation. Acoustic noise is able to reduce TL, but the TP has to be taken into account. Based on our findings, we speculate about a possible future treatment of tinnitus by near-threshold bandpass filtered acoustic noise stimulation, which could be implemented in hearing aids with noise generators.

## Introduction

The most successful therapies for tinnitus usually rely on psychosomatic coping strategies (Malouff et al., 2011; Grewal et al., 2014; Beukes et al., 2018) as well as on cognitive behavioral or tinnitus retraining therapies (Makar et al., 2017; Teixeira, 2018; Fuller et al., 2020) but rarely on physiological approaches. Nevertheless, some recent physiological approaches include deep brain (Streppel et al., 2006) or vagus nerve stimulation (Engineer et al., 2011; Tyler et al., 2017), non-invasive approaches include notched music (Pantev et al., 2012) or desynchronizing acoustic stimulation (Tass et al., 2012) or simply masking the percept with noise (Aytac et al., 2017). Most of these methods may lead to a reduction of tinnitus related distress – dependent on their used questionnaire (Kennedy et al., 2004) – between 10 and 20%. Nevertheless, a single study reports success of up to 50% (Tass et al., 2012). One alternative method that is not primarily a tinnitus treatment but has success reported in several studies in between 50 and 75% of the cases is the implantation of a cochlear implant and therefore partial restoration of hearing itself (e.g., Távora-Vieira et al., 2013). This surgery is performed only in cases of severe hearing impairment and is therefore not suited for the majority of tinnitus patients.

Based on our physiological model of tinnitus development (Krauss et al., 2016; Schilling et al., 2021) – which may be only valid for tinnitus development based on cochlear defects – we are currently developing a new treatment strategy, especially for tinnitus patients without or with only mild hearing loss (HL). This strategy is based, first, on our hypothesis that tinnitus is a byproduct of a neurophysiological mechanism that permanently optimizes information transmission into the auditory system by means of stochastic resonance (SR) – a mechanism well described in other neuronal systems (Douglass et al., 1993; Faisal et al., 2008; Mino, 2014). Here the basic idea is that also in the healthy organism the neuronal hearing threshold signal is constantly adapted for optimal information transmission. This can be achieved by constantly computing the autocorrelation of the neuronal input signal (Krauss et al., 2016). In the case of hearing, this adaptation on the signal level is thought to be achieved by adding neuronal noise to the early stage neuronal signal coming from the cochlea in a frequency specific manner. The added noise intensity is self-regulating, as to much noise decreases information transmission and will therefore be down regulated. We have proposed that the neuronal generated noise is added to the cochlear input at the second synapse, i.e., at the level of the dorsal cochlear nucleus (DCN), thereby lifting neuronal signals above the response threshold of the postsynaptic neuron that would otherwise not respond. With this idea in mind, we propose that when a HL occurs, e.g., by damage to the inner hair cells of the cochlea or the auditory nerve fibers loss independent if it is either clinical detectable or “hidden” HL (Liberman et al., 2015), the information transmission in the affected frequency range is reduced. This reduction is detected by the neuronal system by a reduction of the described autocorrelation, leading to an increase (e.g., by reducing neuronal inhibition) in neuronal noise. As indicated above, such SR would then result in an increased amount of information at the DCN output (Douglass et al., 1993; Faisal et al., 2008; Mino, 2014; Liberman et al., 2015; Krauss et al., 2016, 2017, 2019; Schilling et al., 2021). In the view of our hypothesis, the internal neuronal noise is permanently adjusted at a millisecond timescale to meet the environmental conditions of the auditory scenery, thereby optimizing information transmission constantly. The addition of noise in the case of HL leads to a better detection threshold of the affected frequencies, i.e., recovering the hearing threshold to a certain degree. By propagating the additional noise upstream to the auditory cortex, the signal is there interpreted as a sound – the perceived tinnitus. This idea is strengthened by another recent animal study (Krauss and Tziridis, 2021) where simulating HL by reducing the loudness of specific frequencies – similar to a Zwicker tone (Zwicker, 1964) – leads to a transient tinnitus percept and better hearing thresholds. Further additional support of this view gives the demonstration that tinnitus patients seem to have better hearing thresholds in the – for human communication important – frequency range up to 3 kHz compared to patients without such a phantom percept (Gollnast et al., 2017).

The second basis of our therapeutic approach is the observation that also externally applied near-threshold acoustic noise can improve hearing thresholds in healthy human subjects by up to 13 dB without reports of induced tinnitus percepts (Zeng et al., 2000) – an observation that again can well be explained by the SR mechanism. Our aim was therefore to substitute the internal neuronal noise – which in our view is elevated to overcome a hearing impairment and is perceived as tinnitus – by external near-threshold acoustic noise. The internally generated neuronal noise should therefore become obsolete and should be tuned down, leading to a reduction of tinnitus loudness (TL) or even the complete disappearance of the percept.

Our first pilot study used very crude intensity (−20 dB SL to +20 dB SL in 10 dB steps) but comparable frequency steps to adapt the externally presented noise to the patient’s audiometric data, but it yielded promising results (Schilling et al., 2020). Briefly, we could demonstrate that during the presentation of the stimulation most patients reported a significantly reduced tonal subjective TL – even though we did not use the classical TL measurement of the visual analog scale (Adamchic et al., 2012). The TL reducing effect was only present in patients with a maximal mean hearing impairment of 40 dB. Patients with a HL above this value did not benefit from the approach. Too loud stimulation (≥+10 dB SL) also led in half of the responding patients to masking effects. In other words, only relatively near-threshold stimulation had the desired effect in reducing TL. This led us to the hypothesis (and this study, as an extension of the first pilot work) that with a more fine-grained adaptation of the externally presented noise to the patient’s audiometric data – with respect to both spectrum and amplitude – it should be possible to reduce the subjective tinnitus percept loudness substantially without masking it, at least in patients with a mean HL not exceeding 40 dB.

## Materials and Methods

### Subjects

**FIGURE 1:**
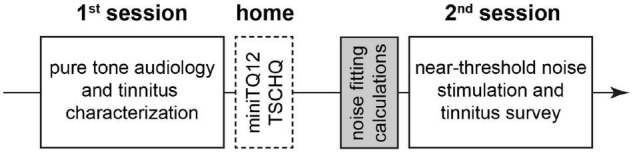
Overview of the temporal sequence of the study. White solid line: interaction with patient during the sessions. White broken line: work of patient at home alone. Gray: preparation of acoustic stimulation by investigator alone.

Twenty-four adult patients (13 women) with a mean age ± standard deviation of 42.9 ± 12.5 years with subjective tonal tinnitus were included in this study with informed consent (University Hospital Erlangen ethics committee vote 159_18B). The patients were specifically recruited for this study by internet and local ENT doctors information leaflets layouts. The main complaint of the patients was the chronic tinnitus percept. As inclusion criterions, the tinnitus had to be tonal and its pitch not above 10 kHz and the maximal HL had to be below 40 dB in the range between 0.5 and 6 kHz. Pure tone hearing thresholds as well as tinnitus pitch (TP) and loudness (TL) between 0.125 and 10 kHz (in some cases audiograms only measured up to 8 kHz) were measured in the audiology department of the ENT hospital Erlangen following ISO 8253-1 procedures. Mean HL was 12.1 ± 6 dB and median TP [interquartile range] was 8 kHz (Pantev et al., 2012; Fuller et al., 2020). If patients reported tinnitus on both sides, the near-threshold noise-parameters (cf. section “Near-Threshold Spectrally Adapted Acoustic Noise”) were fitted to the audiometric data of the ear with the lower HL. Else, the parameters were adjusted to the audiometric data of the tinnitus side. In two cases, both ears were nearly identical, so testing was done for both sides, i.e., we tested 26 individual noise parameters in 24 patients. To exclude patients with decompensated tinnitus we asked everyone to fill out the mini-tinnitus questionnaire miniTQ12 (Hiller and Goebel, 2004); only patients with a maximal severity index (SI) of three (of four) were included in the study. Note that in this study no patient had to be excluded because of this criterion. Additionally, the Tinnitus Sample Case History Questionnaire (TSCHQ in German) (Langguth et al., 2007) was used to evaluate the tinnitus related anamnesis for each patient. For an overview of the timing of all measurements, refer to Figure 1.

### Near-Threshold Spectrally Adapted Acoustic Noise

With the results of the pure tone audiometry and the tinnitus characterization, individually adapted near-threshold spectrally filtered acoustic noise stimuli were generated. Noise intensities ranged from −2 dB SL to +6 dB SL in 2 dB steps, adjusted to the mean hearing level (mean audiogram value in dB SPL of all measured frequencies) of the patient. The types of noises presented included, first, white noise (WN, acoustic range up to 20 kHz). Second, we used five different bandpass (BP, Butterworth filter fourth order) filtered noises with center frequencies ranging from −1 octave below the TP to +1 octave above the TP (maximally up to 10 kHz) in half octave steps and a filter width of ±1/2 octave. The WN and BP noise stimuli frequency domain were comparable to the ones used in our earlier study (Schilling et al., 2020). The third stimulus type was not used before, it was a noise stimulus adjusted to the inverse audiogram (IA). In other words, a noise that is louder at frequencies with larger HL but softer at frequencies with less HL. The overall sound intensities relative to hearing threshold (dB SL) were identical to the ones used in the WN stimulus. These seven different noise types with five intensities each were generated by a custom made Python program (Python 3.6 with Numpy library; Anaconda distribution, Anaconda, Berlin, Germany) and saved on a laptop for later presentation (cf. Schilling et al., 2020). Additionally, one silent stimulus was generated and presented as a control to rule out “imaginary” effects reported by the patients. Note, that this control stimulus did not evoke any change in TL (cf. section “Results”). The patients did not know, when which stimulus was presented.

### Stimulation and Response Recording

Similar to the procedures in the first pilot study (Schilling et al., 2020), the patients were seated in an acoustic chamber and received the acoustic stimulation *via* auditory headphones. The experiments started always with the WN stimuli from lowest to highest intensity, followed by the silent control stimulus and the different BP and the IA noises in the same intensity order. Each stimulus was presented for 40 s and was followed by the experimenter asking the patient if and how her/his perception of the TL changed during stimulation. The patients were instructed to respond with one of five possible answers regarding the change of perceived TL. This response was a number ranging from −2 to +2 with the corresponding meaning (translation from German): “tinnitus became significantly louder” (−2), “tinnitus became somewhat louder” (−1), “no change in TL” (0), “tinnitus became somewhat softer” (+1), and “tinnitus became significantly softer” (+2). The +2 value included cases, where patients reported complete silencing of their tinnitus percept during stimulation (6/24 patients), this was stated by them. Additional information were given and registered, like possible masking, changes in TP or other changes in perception. One complete set of measurements (36 trials) had a duration of 45–60 min and could be paused by the patient at any time. This option was used only occasionally. After the measurement, patients were compensated for their time with fifty Euro.

### Statistical Evaluation

**FIGURE 2:**
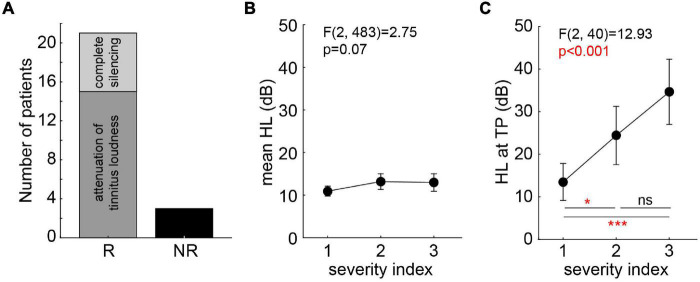
Tinnitus patients’ categorization and severity indices. **(A)** Categorization of tinnitus patients according to their responses during stimulation (R, responder; NR, non-responder). R patients are separated for those with attenuation of tinnitus loudness only (*N* = 15) and those with complete silencing (*N* = 6). **(B)** One-factorial ANOVA of mean HL dependent on miniTQ12 severity index. **(C)** One-factorial ANOVA of HL at TP dependent on miniTQ12 severity index. Results of Tukey *post hoc* tests: ns not significant; *p < 0.05; ***p < 0.001.

Non-parametric statistics was used for the evaluation of the patients’ responses during near-threshold noise stimulation. Based on the same criterion as in our earlier study (Schilling et al., 2020), patients that did not show any positive responses (+1 or +2) to at least one of the 36 stimuli were classified as non-responders (NR, *N* = 3), all other patients were classified as responders (R, *N* = 21; cf. Figure 2A). No R patient showed in only one frequency-intensity combination a response greater than zero, most responders had a “region of best response” spanning at least two neighboring presentation frequencies and/or two to three intensities. Note that by patients’ request (two of the three NR patients) louder than standard BP stimuli were tested at the TP (10 dB SL and 13 dB SL); both NR patients only reported a masking effect at these intensities. Best responses were defined as the highest response (either +1 or +2) at the lowest intensity and frequency of a given BP noise stimulus. Additionally to the individual responses of the patients to each stimulus, the sum of all responses at all intensities of one given stimulus was calculated as a stimulus score. This ranged from a value of −10 to +10, with −10 indicating all stimuli being strongly increasing TL (five times −2) and +10 indicating all stimuli strongly decreasing TL (five times +2). This was also done for the responses obtained in the pilot study (i.e., new analysis of those data) to compare the responses of both studies. The stimulus scores were analyzed using paired non-parametric statistics.

The evaluations of the miniTQ12 and TSCHQ were correlated to the results of the audiometry by parametric (HL) and non-parametric statistics (frequency). Finally, the HL of all ears (*n* = 48) or tinnitus ears only (*n* = 37) were parametrically assessed by one- and two-factorial ANOVAs either with one of the factors being *stimulation frequency* and/or *distance to the TP* in octaves.

## Results

### Interaction of Questionnaire Results and Audiometry

The evaluation of the miniTQ12 resulted in the classification of the patients into all three severity indices included in the study: SI 1: *N* = 13; SI 2: *N* = 6; SI 3: *N* = 5. The overall mean HL was not dependent on the SI [one-factorial ANOVA of HL over *SI*: *F*(2,483) = 2.75, *p* = 0.07; Figure 2B] while the HL at the TP was strongly dependent on the SI [*F*(2,40) = 12,93, *p* < 0.001; Figure 2C] where patients with a SI 1 showed the least strongest HL at the TP with a mean ± standard deviation of 13.5 ± 4.2 dB, the patients with SI 2 following at 24.4 ± 4.5 dB and the SI 3 patients showing the strongest HL at TP of 34.6 ± 12.8 dB. Neither TP [Kruskal–Wallis ANOVA of TP over *SI*: *H*(2,43) = 2.29, *p* = 0.32] nor TL [one-factorial ANOVA of TL over *SI*: *F*(2,39) = 0.65, *p* = 0.53] were dependent on the SI: all patients showed a similar TP and TL ranging from 6 to 8 kHz and −1.4 dB SL to +1.6 dB SL, respectively.

The correlations of the TSCHQ data with the audiometric results showed that neither TP (multiple linear regressions: *r* = −0.30, *p* = 0.15) nor TL (multiple linear regressions: *r* = 0.32, *p* = 0.13) were correlated with the tinnitus duration. The same was true when comparing TL with the subset results of general psychological stress [one-fact. ANOVA of TL over *stress index*: *F*(3,20) = 2.59, *p* = 0.08] and general physical stress [*F*(5,18) = 0.35, *p* = 0.88], indicating that these factors did not influence the TL here.

### Hearing Loss

**FIGURE 3:**
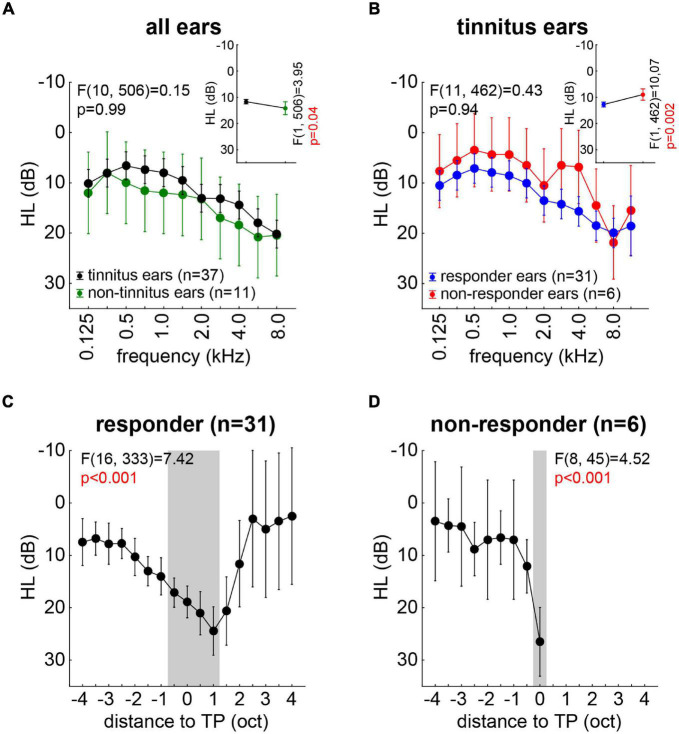
Overview of hearing loss (dB) for all measured ears with F statistics. **(A)** Interaction plot of the two-factorial ANOVA of HL for tinnitus and non-tinnitus ears across all frequencies. The inset depicts the mean HL across all frequencies for both ear types. **(B)** Interaction plot of the two-factorial ANOVA of HL for tinnitus ears only in responders and non-responders. The inset depicts the mean HL for both patient groups. **(C)** One-factorial ANOVA of HL of all responders’ ears aligned on the individual TP. Gray area indicates significant HL revealed by Tukey *post hoc* tests. **(D)** One-factorial ANOVA of HL of all non-responders’ ears aligned on the individual TP. Gray area indicates significant HL revealed by Tukey *post hoc* tests.

Hearing loss was analyzed, first, by two-factorial ANOVAS investigating possible differences between *tinnitus (T) and non-tinnitus (NT) ears* over the *frequency range* of 125–8,000 Hz, as this was the range in that all patients were tested. We found (Figure 3A, inset) a significantly higher HL in the NT (14.2 ± 2.8 dB) compared to the T (11.7 ± 0.8 dB) ears [*F*(1,506) = 3.95, *p* = 0.04]. Additionally, a significant dependency between HL and frequency [*F*(10,506) = 3.90, *p* < 0.001] was found, but no interaction of both factors [Figure 3A; *F*(10,506) = 0.15, *p* = 0.99]. In other words, the patients did hear better with their T ears across all frequencies.

In a second step, we investigated the T ears only and compared the HL of those of the *responders (R)* and the *non-responders* (*NR*; cf. section “Materials and Methods”) over the stimulation *frequencies*. The results are depicted in Figure 3B, with the R patients showing a significantly [inset: *F*(1,462) = 10.07, *p* = 0.002] higher HL (12.4 ± 0.8 dB) compared to the NR patients (8.8 ± 2.0 dB). Again, we found a significant dependency of the HL on the frequency [*F*(11,462) = 5.72, *p* < 0.001] but no interaction of both factors [Figure 3B; *F*(11,462) = 0.43, *p* = 0.94]. This indicated again a parallel shift of the hearing thresholds across all frequencies, this time in favor of the NR patients.

In a third and final step, we aligned the individual HL to the individual TP of each ear and analyzed R and NR ears separately by one-factorial ANOVAs. Figure 3C depicts the results for the R patients’ ears with a significant dependency of the HL on the distance to TP [*F*(16,333) = 7.42, *p* < 0.001]. The Tukey *post hoc* tests revealed that the HL was maximal in a range of −0.5 oct to +1 oct relative to the TP (gray area in Figure 3C). In the NR patients’ ears, we found a similar significant dependency of the HL on the distance to TP [*F*(8,45) = 4.52, *p* < 0.001], but were only able to analyze data up to the TP due to the distribution of the individual tinnitus pitches (Figure 3D). Here, only the HL at the TP was significantly different from the other HL values (Tukey *post hoc* tests, *p*-values between *p* < 0.001 and *p* = 0.03).

### Responses to Near-Threshold Noise Stimulation in Responders

**FIGURE 4:**
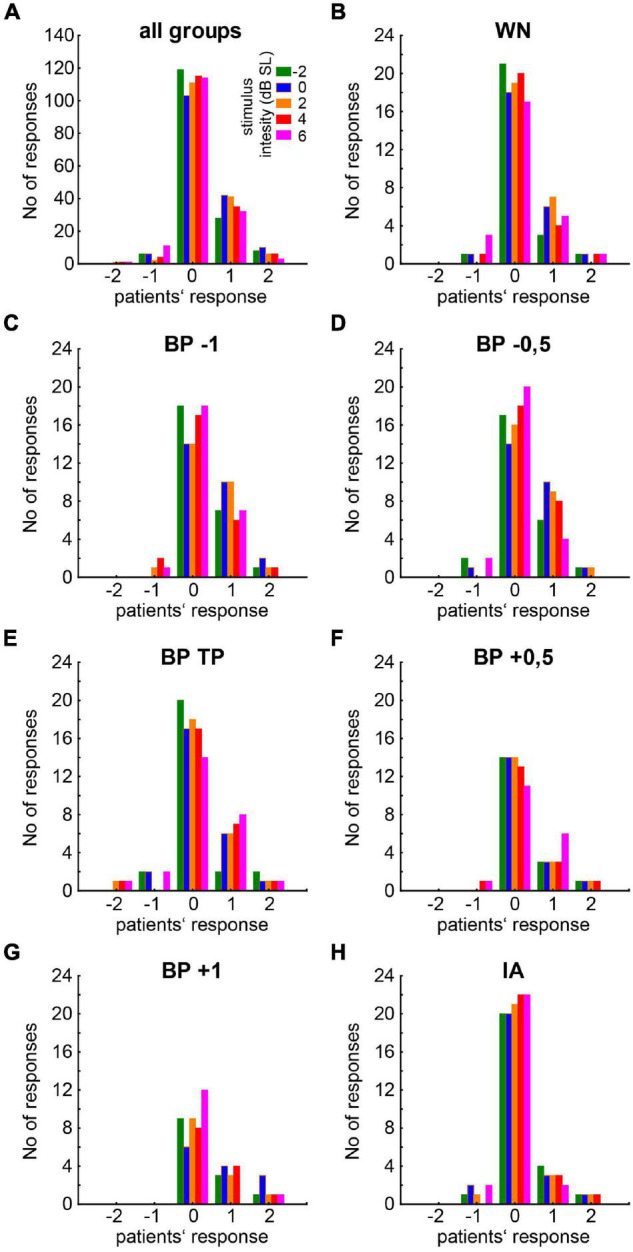
Histogram of patients’ responses to the different stimuli. **(A)** General overview for all stimuli combined. Colors of the bars indicate the noise intensity of the presented stimulus ranging from –2 to 6 dB SL. **(B–H)** Responses to the different isolated stimuli types (WN, BP noises relative to TP, IA).

**FIGURE 5:**
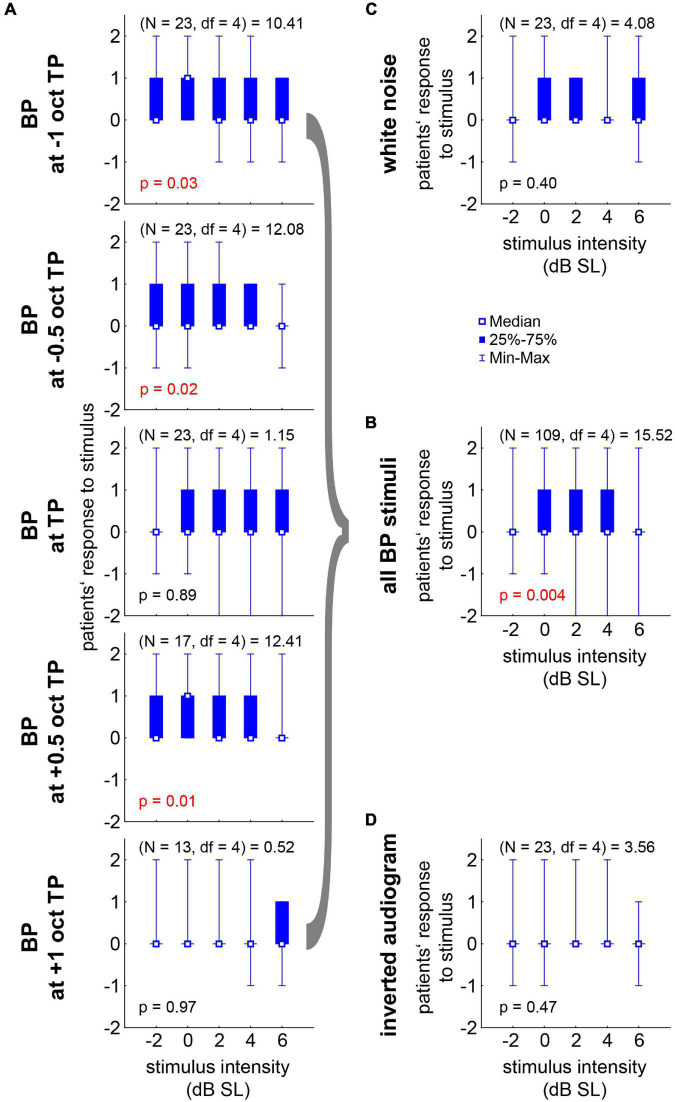
Median responses (–2 to +2 in steps of one) to the near-threshold noise stimuli of all 21 R patients with Friedman-ANOVA statistics. **(A)** Responses to the five BP stimuli ranging from –1 oct to +1 oct relative to TP across the five stimulus intensities. **(B)** Median responses across all BP stimuli. **(C)** Responses to WN stimuli. **(D)** Responses to the IA stimuli.

For an overview of the responses of all patients to the different stimulus conditions (filter type and intensity), please refer to Figure 4. Per definition, the responses of the NR patients never exceeded zero (cf. section “Materials and Methods”) and were therefore not included in the following analyses. From the 21 R patients, 23 datasets were obtained, as two patients had very similar HL on both sides (cf. section “Materials and Methods”) and were therefore tested in both slightly different tinnitus percepts. The median [interquartile range] response values for all R and NR patients are given as overview in Table 1. The median R responses to the noise stimuli were significantly different from the silence stimulus response in all seven cases [Bonferroni corrected Wilcoxon tests: five times *p* < 0.001; one time (BP at TP) *p* = 0.004; one time (IA) *p* = 0.02]. A graphical overview of the median responses of the R patients is given in Figure 5. For each BP filtered noise, a Friedman ANOVA over the five different *stimulus intensities* was calculated (Figure 5A). A significant dependency of the responses on the intensity was found at −1 oct, −0.5 oct, and +0.5 oct relative to TP, i.e., in three of the five BP filtered noise stimulus frequencies. For a better overview, the median values of all five BP filtered noises have been combined and compared across the five different intensities by a separate Friedman ANOVA (Figure 5B), showing a significant (*p* = 0.004) dependency of the responses on the stimulus intensity, seemingly centered around +2 dB SL. Such dependencies could not be found for the WN stimulus (Figure 5C) or the IA stimulus (Figure 5D).

**TABLE 1:** Median responses [interquartile range] to noise stimuli of R and NR patients.

Stimulus	Responder					Non-responder				
	Stimulus intensity (dB SL)					Stimulus intensity (dB SL)				
	−2	0	+2	+4	+6	−2	0	+2	+4	+6
Silence		0 [0, 0]					0 [0, 0]			
WN	0 [0, 0]	0 [0, 1]	0 [0, 1]	0 [0, 0]	0 [0, 1]	0 [0, 0]	0 [−1, 0]	0 [0, 0]	0 [−1, 0]	0 [0, 0]
IA	0 [0, 0]	0 [0, 0]	0 [0, 0]	0 [0, 0]	0 [0, 0]	0 [0, 0]	0 [0, 0]	0 [−1, 0]	0 [0, 0]	0 [0, 0]
BP −1	0 [0, 1]	1 [0, 1]	0 [0, 1]	0 [0, 1]	0 [0, 1]	0 [0, 0]	0 [0, 0]	0 [0, 0]	0 [0, 0]	0 [0, 0]
BP −0.5	0 [0, 1]	0 [0, 1]	0 [0, 1]	0 [0, 1]	0 [0, 0]	0 [0, 0]	0 [0, 0]	0 [0, 0]	0 [0, 0]	0 [0, 0]
BP TF	0 [0, 0]	0 [0, 1]	0 [0, 1]	0 [0, 1]	0 [0, 1]	0 [−1, 0]	0 [0, 0]	0 [0, 0]	0 [0, 0]	0 [0, 0]
BP +0.5	0 [0, 1]	1 [0, 1]	0 [0, 1]	0 [0, 1]	0 [0, 0]	0 [0, 0]	0 [0, 0]	0 [0, 0]	0 [0, 0]	0 [0, 0]
BP +1	0 [0, 0]	0 [0, 0]	0 [0, 0]	0 [0, 0]	0 [0, 1]					

**FIGURE 6:**
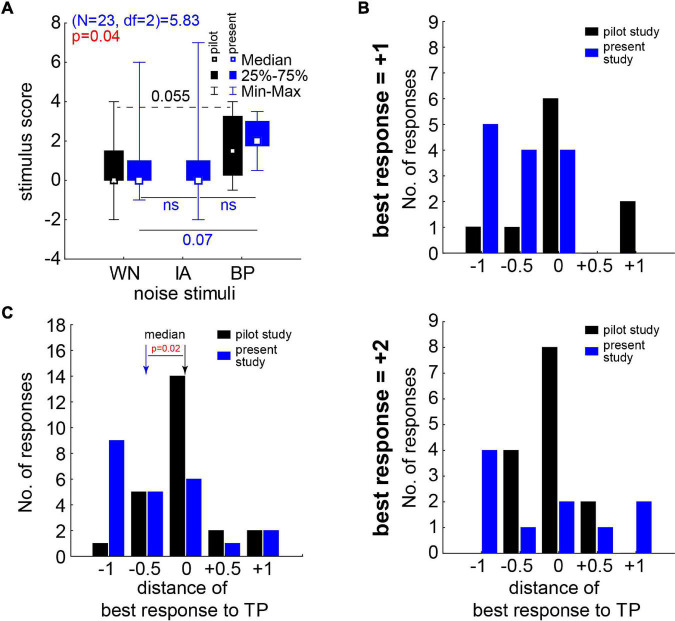
Stimulus score and best responses in comparison to our first pilot study’s data (Schilling et al., 2020). **(A)** Median stimulus score of the pilot study (black symbols and numbers) and this study (blue/red symbols and letters/numbers). Pilot study statistics with Wilcoxon test (broken line); this study with Friedman ANOVA and Wilcoxon tests (solid lines) corrected for repeated comparisons. **(B)** Number of best responses in both studies dependent on the distance to TP of the center frequency of the BP noises. Upper panel: results for best response of +1. Lower panel: results for best response of +2. **(C)** Complete distributions of best responses in both studies; median of both studies significantly different (Mann–Whitney U test).

Finally, in Figure 6 we compared the overall stimulus score and the best responses (cf. section “Materials and Methods”) obtained in the first pilot study with the less fine-grained paradigm with stimuli intensities ranging from −20 to +20 dB SL (Schilling et al., 2020) with those obtained in the present study. For the stimulus score of the different noise stimuli in this study, the significant Friedman ANOVA (*p* = 0.04, Figure 6A, blue symbols) indicated a stronger effect for at least one class of stimuli. The Bonferroni corrected *post hoc* Wilcoxon tests showed a trend (*p* = 0.07) for a higher median score during BP stimulation compared to the WN stimulus responses. No significant difference could be found between WN and IA scores. The effect in the pilot study (black symbols) was somewhat smaller compared to the present study, as the direct comparison of WN and BP noise stimuli by a Wilcoxon test (without correction for multiple comparisons) only showed a trend (*p* = 0.055). Nevertheless, neither in WN nor in BP noise stimuli the Mann–Whitney U tests showed differences between the response scores of both studies (Figure 6A; black vs. blue symbols, *p* > 0.05 in both tests). Also in the distributions of the best responses (Figure 6B), no significant difference between both studies could be found for both best response types of either +1 (Kolmogorov–Smirnov test, *p* = 0.82) or +2 (Kolmogorov–Smirnov test, *p* = 0.80). But the median best responses relative to TP (Figure 6C) were significantly shifted to lower frequencies (Mann–Whitney U test, *p* = 0.02) in the present study (−0.5 [−1, 0] oct TP) compared to the first pilot study (0 [−0.5, 0] oct TP). This indicates that the near-threshold stimuli used in this study were effective at lower frequencies relative to TP compared to the much louder stimuli used in the first pilot study.

## Discussion

In this extension study of our pilot work, we aimed to further narrow down parameters for near-threshold acoustic stimulation with individually filtered soft noises to attenuate or even silence tinnitus perception during stimulation. Based on our hypothesis of tinnitus development due to a SR mechanism for optimization of auditory information transfer, we applied near-threshold individually adapted acoustic noise *via* headphones to 24 tinnitus patients. This approach is not comparable with the classic “tinnitus noiser” (Zenner et al., 2017), as it is not aimed to mask the phantom percept but to attenuate or ideally cancel it by assessing its physiological cause. In the previous pilot study (Schilling et al., 2020), we found in half of the responding patients masking effects when exceeding +10 dB SL stimulation loudness. This was not the case in the present work as we focused on stimuli not exceeding +6 dB SL. Note that in the two cases were we exceeded this intensity on patients’ request, masking effects were reported at +10 and +13 dB SL.

In 21 of the investigated 24 tinnitus patients (nearly 88%) without or only mild HL, this approach was successful – at least on a subjective level. Six of those 21 responding patients (nearly 29%) even reported complete subjective silencing of their tinnitus percept during stimulation. The HL in the tinnitus ears of all 24 patients was significantly lower than the HL in the NT ears, which is completely in line with our hypothesis that SR improves the hearing thresholds on the cost of generating tinnitus and supported by data of a large patient cohort (Krauss et al., 2016; Gollnast et al., 2017). The three patients not responding to the near-threshold acoustic stimulation showed significantly lower HL than the 21 responding patients, indicating that we may not only have an upper HL limit of around 40 dB for successful stimulation (cf. Schilling et al., 2020) but also a lower HL limit. In this case, we are maybe still too loud, and in future studies even softer stimuli below −2 dB SL should be used in such patients. Alternatively, these patients might not have a HL at all but may suffer from a different kind of tinnitus source, as for example stress (Mazurek et al., 2015) or other non-auditory reasons (Bauer, 2004). This may explain why the modulation of auditory input has no or only a masking effect on the tinnitus percept. The optimal noises for the 21 subjectively responding patients were in all cases bandpass filtered stimuli with an intensity between 0 and +4 dB SL (Figure 5B) and a best noise center frequency of half an octave below the individual TP (Figure 6C). WN or noise filtered with the characteristics of the IA did not have these consistent positive effects on the subjective percepts. This could be due to the wide spectrum of these kind of stimuli. WN as well as the IA noise to a certain degree stimulate the whole cochlea, while the BP noise stimuli stimulate only specific cochlear regions with acoustic energy “focused” to or close to the TP. These physical differences in stimulation combined with our hypothesis of frequency channel specific SR (Krauss et al., 2019) suggests that only stimulation in the “correct” frequency range will have positive effects on perceived subjective TL. The situation could be different, e.g., in patients with non-tonal tinnitus percepts and has to be investigated in a separate follow-up study with such patients. Taken together, these findings might be very important for future adaptation of, e.g., hearing aids with noisers (cf. below), as a shift in TP may need adjustment in stimulation frequency, which could be performed by the patients themselves when provided with the adequate software tool.

On the other hand, the here presented results in combination with our hypothesis of the SR mechanism for tinnitus development also shows clear limitations of our method. First, it seems to work only in a relatively narrow HL window, most probably because the SR mechanism is only able to compensate for a certain degree of hearing impairment. Additionally, it seems that the mechanism is not working in all humans identically well – which is also supported by results from animal research (Ahlf et al., 2012). Also the type of hearing impairment seems to play an important role, as not all HL patients with different kinds of hearing impairment have tinnitus or show specific hearing threshold benefits due to their tinnitus percept (Gollnast et al., 2017). Second, the SR mechanism only explains the bottom-up generation of the tinnitus signal, not the different top-down influences coming from, e.g., the amygdala, higher cortical areas or even the back-projections from the cortex to the auditory brainstem. This may also explain the conflicting results of the (missing) correlations of TL or severity with the hearing threshold loss in different studies (e.g., Searchfield et al., 2007; Mazurek et al., 2010) which cannot be explained by the SR mechanism alone. In other words, the proposed approach to dampen perceived TL is most probably not able to help all patients, but should at least be helpful for patients with maximally mild HL and compensated tonal tinnitus. Here, the main driving force of the percept is in our view the increased noise from the auditory brainstem.

One has to be careful to disentangle TL from tinnitus distress. It could be shown that both aspects of the percept are not necessarily directly linked (Hiller and Goebel, 2007; Wallhäußer-Franke et al., 2012) so even if we can dampen the one, it might not affect the other. In the 40 s approach of both our studies, we were not able to measure the distress and only crudely the TL, as we did not use, e.g., the visual analog scale (Adamchic et al., 2012). This has to be included in studies with longer stimulation duration. Nevertheless, several patients mentioned that they were relieved when it became clear that we were able to dampen their TL. Most patients were provided with “their” optimal stimulus for playing on a mobile device and – anecdotic – we received messages from two patients reporting long-term success and strong subjective relieve of their distress.

When comparing the here presented results with the first pilot study with overall 22 patients (Schilling et al., 2020) we see, first, that we have a comparable (maybe slightly stronger) positive effect of the bandpass filtered noises on the subjective suppression of the TL with the current stimulation parameters (cf. Figure 6A). Second, we see a significantly lower best response center frequency of the noise in this study compared to the first pilot study (cf. Figure 6C). If this finding is consistent in follow-up studies, it makes it easier to stimulate in the long term, e.g., with specifically adapted hearing aids with noise generators (Del Bo and Ambrosetti, 2007). As these stimulation frequencies would be just at the edge of the significant HL of the patient collective (cf. Figure 3C), they should be soft enough to be adjusted correctly and not harmful in any way for the patients’ hearing. Third, with the here used stimuli of much lower intensity compared to those of the first pilot study we have a significantly lower (Chi-square test, *p* = 0.008) fraction of masking. While in the first study 50% of the responders reported masking effects mainly at +10 and +20 dB SL, in this study only 9% (two of the NR patients at +10 dB SL and +13 dB SL, respectively) of all patients and none of the responding patients reported such a percept. On the other hand, we did not find a difference of the strength of the reported subjective decrease in subjective TL (Chi-square test, *p* > 0.05) as in the first pilot study 58% and in this study 48% of the patients reported a strong decrease (+2) in this parameter. In both studies, we used only a single control stimulus – silence – to control for a placebo effect and a fixed presentation order of the stimuli. As the patients did not know when which stimulus would be presented, each patient had her/his individual stimulus design and each patient was only tested once, any order effect should be minimal but cannot be ruled out completely. In the placebo silence test, not a single patient indicated a subjective change in TL – neither positive nor negative – in the overall 48 presentations, indicating that no stimulation also has no effect. This is clearly different from other studies showing up to 40% placebo effect (Duckert and Rees, 1984). A weakness of both of our studies is that we lack a true control group, this would strengthen the points mentioned above and is planned to be included in any follow-up studies. Nevertheless, our simple approach of asking the patients after each short test can only be a first step in investigating the possible therapeutic effect of the individualized noise exposure against tinnitus. Further experiments with healthy controls and longer noise exposition with adapted hearing aids with noise generators and objective tests and questionnaires are already planned.

As this approach for the development of a physiological treatment for tinnitus is unique, it is difficult to compare it with other methods of tinnitus therapies. Even though, several other groups also found that the TP has a strong influence on hearing and hearing aids (McNeill et al., 2012; Haab et al., 2019; Shetty and Pottackal, 2019), which is in line with our findings and hypothesis, most of these researchers tried completely different approaches like specific masking with the help of a device. Our approach is clearly different from this classical “noiser” or sound generator approaches, as the intensities used here are just at the level or slightly above the hearing thresholds. Classical “noisers” are using much higher intensities to mask the percept successfully, but on the cost of the noise being permanently perceived. In other words, one sound (tinnitus) is replaced by another sound (noise). Furthermore, the effects found here cannot be explained by residual inhibition, as this takes effect at intensities of +10 dB minimum masking level and takes several minutes of constant stimulation (King et al., 2021). In our case, the effect was immediate at +2 dB SL, i.e., within a few seconds after stimulation start and lasting only until the end of the stimulation. Also lateral inhibition, as used in different notch filter approaches (Haab et al., 2019), would not be able to explain the observed effects, as also here the used sound intensities and time scales are much larger and the filter properties are inverted relative to our approach. The success rate of our method of up to 87.5% in at least reducing the subjective TL is only comparable with the up to 75% rate of tinnitus suppression by cochlear implants (Távora-Vieira et al., 2013). Both methods are completely different in the mechanisms addressed, while we proposedly modulate the neuronal SR mechanism by external acoustic stimulation in mostly well hearing patients, the implantation of the neuroprosthetics enables the cochlear nerve to receive information again and thereby restores hearing in formerly deaf regions of the cochlea. In other words, the target patient cohorts for these two methods are on the opposite spectrum of hearing impairments.

## Conclusion

In conclusion, the hypothesis of the present study that the proposed treatment would reduce subjective TL in all patients with maximally mild HL was not confirmed as only around 88% of the individuals benefited from it. The present study indicates strong need for a randomized placebo-controlled study of the proposed treatment in order to clearly determine possible benefits of the treatment. One could speculate that tinnitus patients without or only mild HL, who usually would not be supplied with a classical hearing aid, may profit strongly from such a device when it is equipped with a noise generator that produces the right amount of individually adjusted near-threshold noise in the right frequency range.

## Data Availability Statement

The raw data supporting the conclusions of this article will be made available by the authors, without undue reservation.

## Ethics Statement

The studies involving human participants were reviewed and approved by the University Hospital Erlangen Ethics Committee vote 159_18B. The patients/participants provided their written informed consent to participate in this study.

## Author Contributions

KT and AS planned the experiments. SB performed all experiments. KT, PK, and HS wrote the manuscript. KT and SB performed the statistical analyses. AS provided the software and assisted with technical expertise. PK had substantial contributions to the interpretation of data, revised the manuscript critically, especially the discussion part, approved the final version for publication, and agreed to be accountable for all aspects of the work in ensuring that questions related to the accuracy or integrity of any part of the work are appropriately investigated and resolved. All authors contributed to the article and approved the submitted version.

## Conflict of Interest

The authors declare that the research was conducted in the absence of any commercial or financial relationships that could be construed as a potential conflict of interest.

## Publisher’s Note

All claims expressed in this article are solely those of the authors and do not necessarily represent those of their affiliated organizations, or those of the publisher, the editors and the reviewers. Any product that may be evaluated in this article, or claim that may be made by its manufacturer, is not guaranteed or endorsed by the publisher.

## Abbreviations

DCN: dorsal cochlear nucleus
HL: hearing loss
IA: inverse audiogram noise
miniTQ12: mini Tinnitus Questionnaire with 12 questions (German version)
NR: non-responder
NT: non-tinnitus
R: responder
SI: severity index
SL: sensation level
T: tinnitus
TP: tinnitus pitch
TL: tinnitus loudness
TSCHQ: Tinnitus Sample Case History Questionnaire (German version).
